# ZFP90 drives the initiation of colitis-associated colorectal cancer via a microbiota-dependent strategy

**DOI:** 10.1080/19490976.2021.1917269

**Published:** 2021-05-05

**Authors:** Ji-Xuan Han, Zhi-Hang Tao, Yun Qian, Chen-Yang Yu, Jialu Li, Zi-Ran Kang, Shiyuan Lu, Yuanhong Xie, Jie Hong, Haoyan Chen, Ying-Xuan Chen, Jing-Yuan Fang

**Affiliations:** State Key Laboratory for Oncogenes and Related Genes, Key Laboratory of Gastroenterology and Hepatology, Ministry of Health, Division of Gastroenterology and Hepatology, Shanghai Institute of Digestive Disease, Renji Hospital, School of Medicine, Shanghai Jiao Tong University, Shanghai, China

**Keywords:** ZFP90, gut microbiota, colitis-associated colorectal cancer, gut barrier, *prevotella copri*

## Abstract

Chronic inflammation and gut microbiota dysbiosis are risk factors for colorectal cancer. In clinical practice, patients with inflammatory bowel disease (IBD) have a greatly increased risk of developing colitis-associated colorectal cancer (CAC). However, the underlying mechanism of the initiation of CAC remains unknown. Systematic analyses using an existing genome-wide association study (GWAS) and conditional deletion of *Zfp90* (encoding zinc finger protein 90 homolog) in a CAC mouse model indicated that *Zfp90* is a putative oncogene in CAC development.

Strikingly, depletion of the gut microbiota eliminated the tumorigenic effect of *Zfp90* in the CAC mouse model. Moreover, fecal microbiota transplantation demonstrated that *Zfp90* promoted CAC dependent on the gut microbiota. Analysis of 16s rDNA sequences in fecal specimens from the CAC mouse model allowed us to speculate that a *Prevotella copri*-defined microbiota might mediate the oncogenic role of *Zfp90* in the development of CAC. Mechanistic studies revealed *Zfp90* accelerated CAC development through the TLR4-PI3K-AKT-NF-κB pathway. Our findings revealed the crucial role of the *Zfp90*-microbiota-NF-κB axis in creating a tumor-promoting environment and suggested therapeutic targets for CAC prevention and treatment.

## Introduction

Colorectal cancer (CRC) ranks as the third most common cancer and the second leading cause of cancer-related death worldwide.^1,2^ The development of CRC is influenced by multiple factors, which include genetic predisposition, dysbiosis of the gut microbiota, and environmental factors.^3,4^ Previous research showed that chronic inflammation could accelerate the development of CRC.^5^ In the clinical scenario, patients with inflammatory bowel disease (IBD) are predisposed to CRC, and colitis-associated colorectal cancer (CAC) has been recognized as a challenging complication in this population.^6,7^ A fundamental understanding of the evolutionary process underpinning carcinogenesis is vital to optimize the management of CAC risk in patients with IBD.

Genome-wide association study (GWAS) is an observational study of a genome-wide set of genetic variants in different individuals that allows the identification of disease susceptibility variants. Results from GWAS have been overlapped to identify potential pluripotent genes that are involved in diseases such as obesity and cardiovascular disease.^8–10^ GWAS and subsequent fine-mapping research have identified more than 50 genetic susceptibility loci for CRC^11^ and more than 240 susceptibility loci for IBD.^12^ Given the well-known association between IBD and CRC, exploring shared molecular pathways between the two diseases by taking advantage of existing GWAS might provide novel therapeutic targets for CAC prevention and treatment.^13^

Accumulating evidence shows that dysfunction of the gut barrier contributes to the development of both IBD and CRC.^14,15^ The monolayered gut epithelium, together with goblet cell-derived mucins and Paneth cell-derived antimicrobial peptides, forms a physical and biochemical barrier that segregates the intestinal content and the host immune system. Breach of intestinal barrier can lead to increased infiltration of molecules such as lipopolysaccharide (LPS), which then abnormally activates the host immune system, resulting in an inflammatory microenvironment. Thus, increased intestinal permeability is emerging as one of the hallmarks of CAC.^15,16^

The microbiota plays a major role in human health and diseases, including CRC and IBD. Many factors, including host genetics and external factors, such as diet, lifestyle, and medication, can affect the composition of the gut microbiota. Host genetics define the chemical and physical landscape in which the gut microbiota resides^17^ and genetic variations between individuals can result in significant differences in the gut microbiota.^18,19^ Many studies have found that the effects of certain genes on disease progression are partly mediated by modulating the intestinal microbiota.^20–23^ Additionally, as part of the gut barrier, the distribution and structure of tight junction proteins have been shown to be modified by the gut microbiota.^24–26^

In the present study, through overlapping findings from GWAS, we found that *ZFP90* (encoding zinc finger protein 90 homolog) is associated with the development of CAC. We verified that deletion of *Zfp90* in the mouse colonic epithelium reduced CAC formation. We showed that *Zfp90*^ΔIEC^ mice (conditional deletion of *Zfp90* in intestinal epithelial cells (IECs)) exhibited a better gut barrier function and milder inflammation upon azoxymethane and dextran sulfate sodium (AOM-DSS) treatment. Furthermore, we confirmed that the protective role of *Zfp90* deletion in AOM-DSS-induced CAC is dependent on the gut microbiota.

## Results

### ZFP90 *is associated with CRC and IBD and* Zfp90*^ΔIEC^ mice are less susceptible to AOM-DSS-induced CAC*

**Figure 1.**
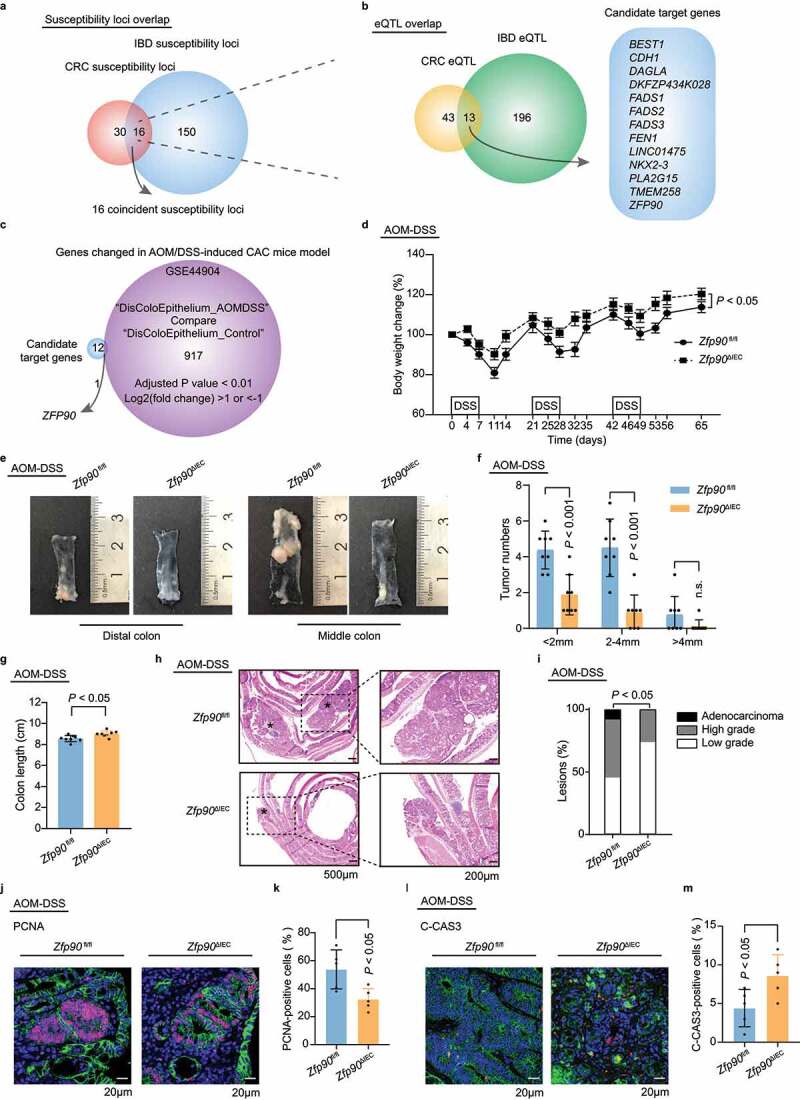
***ZFP90* is associated with CRC and IBD and *Zfp90*^ΔIEC^ mice are less susceptible to AOM-DSS-induced CAC a** Venn diagram showing the chromosome loci accounting for CRC (46 loci), IBD (166 loci), and both (16 loci). **b** Venn diagram showing the genes regulated by CRC-related SNPs (56 genes), IBD-related SNPs (209 genes), and both (13 genes). **c** *ZFP90* was identified after overlapping 13 candidate target genes with genes differentially expressed during AOM-DSS treatment (918 genes). **d** The body weight of *Zfp90*^fl/fl^ and *Zfp90*^ΔIEC^ mice was recorded throughout the experiment and was expressed as the ratio relative to the initial weight before DSS treatment (n = 8 per group). **e** Representative images of distal and middle colons in AOM-DSS-treated *Zfp90*^fl/fl^ and *Zfp90*^ΔIEC^ mice. **f** Tumor distribution in *Zfp90*^fl/fl^ and *Zfp90*^ΔIEC^ mice (n = 8 per group). **g** Colon length in *Zfp90*^fl/fl^ and *Zfp90*^ΔIEC^ mice at the end of the experiment (n = 8 per group). **h** Representative H&E staining of colon tumors from *Zfp90*^fl/fl^ and *Zfp90*^ΔIEC^ mice. * tumors. **i** Proportion of low-grade dysplasia, high-grade dysplasia, and adenocarcinoma in the colons. **j** Representative immunofluorescence staining of the PCNA protein in tumor tissues from *Zfp90*^fl/fl^ and *Zfp90*^ΔIEC^ mice. Sections were stained with DAPI (blue), CDH1 (green) and PCNA (red). **k** The percentage of PCNA-positive tumor cells was quantified (n = 5 per group). **l** Representative immunofluorescence staining of C-CAS3-positive cells in tumor tissues from *Zfp90*^fl/fl^ and *Zfp90*^ΔIEC^ mice. Sections were stained with DAPI (blue), CDH1 (green) and C-CAS3 (red). **m** The percentage of C-CAS3-positive tumor cells was quantified (n = 5 per group). Data with error bars represent the mean ± SD. Each panel is a representative experiment of at least three independent biological replicates. Two-way ANOVA (d, f), nonpaired two-tailed t-test (g, k, m), Fisher’s exact test (i)

To identify a gene that might play a crucial role in the progression of CAC, we first searched HaploReg to explore candidate regulatory single nucleotide polymorphisms (SNPs) for CRC and IBD.^27^ Sixty-nine SNPs responsible for CRC and 386 SNPs responsible for IBD were identified. We then located the susceptibility loci of these SNPs using the UCSC Genome Browser.^28^ In total, 46 CRC susceptibility loci and 166 IBD susceptibility loci were identified. Among them, 16 loci were coincident (Figure 1a). To identify potential target genes regulated by the SNPs at these 16 loci, we performed electronic quantitative trait locus (eQTL) analyses in the Genotype-Tissue Expression (GTEx) portal and identified 13 candidate genes^29^ (Figure 1b). To seek genes that were differentially expressed in the process of AOM-DSS-induced CAC, the expression profile GSE44904 was obtained from the Gene Expression Omnibus (GEO) database.^30,31^ With the thresholds of adjusted *P* < .01 and |log-fold change (FC)| > 1, 918 genes were found to have significantly differential expression between AOM-DSS-induced CAC and the controls (Supplementary SFigure 1a). Next, we combined these differentially expressed genes with the 13 candidate genes mentioned above to discover the genes that are likely to play an important role in the initiation of CAC. After overlapping, *ZFP90* was the only gene that appeared in both clusters (Figure 1c). Expression profile GSE44904 showed that *Zfp90* expression increased during AOM-DSS treatment, indicating a potential oncogenic role of this gene (Supplementary SFigure 1b). Therefore, we chose *Zfp90* as the candidate biological target gene and explored its underlying mechanisms in the development of CAC.

To corroborate the role of intestinal ZFP90 in the development of CAC, *Zfp90* was conditionally knocked out in the mouse IECs to generate IEC-specific *Zfp90* deficient mice (*Zfp90*^ΔIEC^). The morphology of the colonic epithelium was similar between *Zfp90*^fl/fl^ and *Zfp90*^ΔIEC^ mice. (Supplementary SFigure 1c). *Zfp90*^fl/fl^ and *Zfp90*^ΔIEC^ mice were housed separately by genotypes after weaning. Mono-housed male *Zfp90*^fl/fl^ and *Zfp90*^ΔIEC^ mice were then treated with one dose of AOM followed by three cycles of feeding water containing DSS to induce a CAC model in a specific-pathogen-free (SPF) environment (Supplementary SFigure 1d). The *Zfp90*^ΔIEC^ mice exhibited less weight loss than the *Zfp90*^fl/fl^ mice during AOM-DSS treatment (Figure 1d). At the end of the experiment, the whole colon of each mouse was dissected for measurement. Compared with the *Zfp90*^fl/fl^ mice, the *Zfp90*^ΔIEC^ mice showed reduced numbers of colorectal tumors in the distal and middle region, their tumors were smaller (Figure 1(e, f) **and** Supplementary SFigure 1e), and milder colitis was characterized by a longer colon length (Figure 1g). In addition, the spleens were smaller in the AOM-DSS-treated *Zfp90*^ΔIEC^ mice than in the *Zfp90*^fl/fl^ mice, indicating alleviation of systemic inflammation in the *Zfp90*^ΔIEC^ mice (Supplementary SFigure 1f). Although dysplasia was observed in both the *Zfp90*^fl/fl^ and *Zfp90*^ΔIEC^ mice, high-grade dysplasia was more common in the *Zfp90*^fl/fl^ mice. Notably, adenocarcinoma invasion into the submucosa was only observed in the *Zfp90*^fl/fl^ mice (Figure 1(h, i). Proliferative activities were assessed, and reduced proliferation and increased apoptosis were detected in the *Zfp90*^ΔIEC^ mice, as shown by lower percentages of proliferating cell nuclear antigen (PCNA)-positive cells (Figure 1(j, k)) and higher numbers of cleaved caspase3 (C-CAS3)-positive cells (Figure 1(l, m)).

herefore, we explored whether *Zfp90* deletion had an impact on the intrinsic response to inflammatory stimuli. Mono-housed *Zfp90*^fl/fl^ and *Zfp90*^ΔIEC^ mice were given one period of DSS solution for 7 days and then transferred to autoclaved water for 5 days to induce an acute colitis model. Similarly, the *Zfp90*^fl/fl^ mice exhibited more weight loss (Supplementary SFigure 1g) and a shorter colon length (Supplementary SFigure 1h) compared with those of the *Zfp90*^ΔIEC^ mice under DSS-induced acute colitis.

According to our previous study,^32^ a SNP residing in the intron of *CDH1* (encoding cadherin 1) could mediate a long-range regulation on *ZFP90*. Therefore, we could not exclude the possibility that the expression of *CDH1* might also be regulated by *ZFP90* because of their proximity, and such modulation might mediate the protective role of *ZFP90* deletion, considering the importance of *CDH1* in IBD and CRC.^33,34^ We thus tested the expression level of CDH1 in naïve and AOM-DSS-treated *Zfp90*^fl/fl^ and *Zfp90*^ΔIEC^ mice. However, no differences in the mRNA and protein levels of CDH1 were found in colon tissues of the *Zfp90*^fl/fl^ and *Zfp90*^ΔIEC^ mice (Supplementary SFigure 1i, j). This observation suggested that *Cdh1* is not involved in the phenotypic differences between *Zfp90*^fl/fl^ and *Zfp90*^ΔIEC^ mice.

### Zfp90 *deletion in IECs improves the gut barrier and alleviates local inflammation during CAC development*

**Figure 2.**
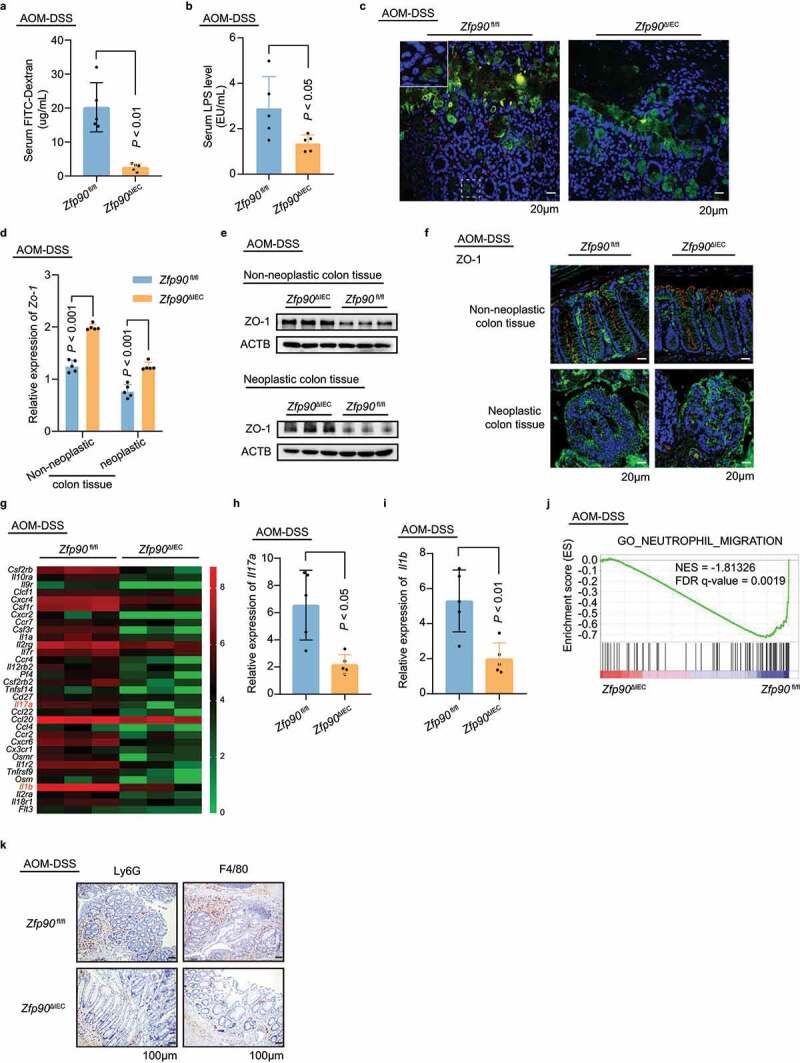
*Zfp90* deletion in IECs improves the gut barrier and alleviates local inflammation during CAC development

Given the importance of barrier dysfunction in the development of IBD and CRC, we evaluated the gut permeability in mono-housed AOM-DSS-treated *Zfp90*^fl/fl^ anfd *Zfp90*^ΔIEC^ mice using fluorescein isothiocyanate (FITC)-dextran. The *Zfp90*^fl/fl^ mice displayed higher gut permeability compared with the *Zfp90*^ΔIEC^ mice under AOM-DSS treatment, as shown by more FITC-dextran leakage into the circulation (Figure 2a). We also assessed gut-derived LPS levels in the serum of AOM-DSS-treated *Zfp90*^fl/fl^ and *Zfp90*^ΔIEC^ mice. Consistently, the *Zfp90*^fl/fl^ mice exhibited markedly increased LPS translocation compared with the *Zfp90*^ΔIEC^ mice (Figure 2b). To determine whether gut microbes can traverse the impaired gut barrier in *Zfp90*^fl/fl^ mice, we performed fluorescence *in situ* hybridization (FISH) using a bacterial 16S probe to visualize the bacteria-mucus-epithelium interface. More bacteria were found to be in direct contact with the epithelial cells, or even reached deep down into the crypts through the mucus in *Zfp90*^fl/fl^ mice compared with those in the *Zfp90*^ΔIEC^ mice (Figure 2c). Tight junctions, as part of the gut barrier composition, connect adjacent IECs and regulate gut permeability.^35^ We found that zona occludens 1 (ZO-1) was expressed at a lower level in both the non-neoplastic and neoplastic colon tissue of the *Zfp90*^fl/fl^ mice treated with AOM-DSS compared with that in the *Zfp90*^ΔIEC^ mice (Figure 2(d, e). Additionally, ZO-1 could be visualized with a tight honeycomb-like staining pattern in the non-neoplastic colon tissue of the *Zfp90*^ΔIEC^ mice. Whereas disorganized structures were observed in the neoplastic colon tissue of *Zfp90*^ΔIEC^ mice and the non-neoplastic colon tissue of *Zfp90*^fl/fl^ mice. Notably, ZO-1 was almost invisible in the neoplastic colon tissue of *Zfp90*^fl/fl^ mice (Figure 2f). Collectively, these results demonstrated that conditional deletion of *Zfp90* in the intestines can alleviate the impaired gut barrier induced by AOM-DSS treatment.

**a** Intestinal permeability assessed by FITC-dextran in AOM-DSS-treated *Zfp90*^fl/fl^ and *Zfp90*^ΔIEC^ mice (n = 5 per group). **b** Serum levels of LPS in AOM-DSS-treated *Zfp90*^fl/fl^ and *Zfp90*^ΔIEC^ mice (n = 5 per group). **c** Representative immunofluorescence staining of the mucus UEA1 lectin and 16S bacterial probe in colon segments from AOM-DSS-treated *Zfp90*^fl/fl^ and *Zfp90*^ΔIEC^ mice. Inset: Magnified image of the area shown by the white dashed box, showing penetration of bacteria into the crypt region. Sections were stained with DAPI (blue), UEA1 lectin (green) and EUB338 universal bacterial FISH probe (red). **d** Real-time PCR was performed to determine the mRNA expression of *Zo-1* in both neoplastic and non-neoplastic colon tissues from AOM-DSS-treated *Zfp90*^fl/fl^ and *Zfp90*^ΔIEC^ mice (n = 5 per group). **e** Representative data showing protein level of ZO-1 in both neoplastic and non-neoplastic colon tissues from AOM-DSS-treated *Zfp90*^fl/fl^ and *Zfp90*^ΔIEC^ mice. **f** Representative immunofluorescence staining of the ZO-1 protein in colon segments from AOM-DSS-treated *Zfp90*^fl/fl^ and *Zfp90*^ΔIEC^ mice. Sections were stained with DAPI (blue), CDH1 (green) and ZO-1 (red). **g** Heatmap representing the log_2_-normalized count of cytokine-cytokine receptor interaction pathway expression in the colonic epithelium from *Zfp90*^fl/fl^ and *Zfp90*^ΔIEC^ mice (n = 3 per group). **h, i** Real-time PCR was performed to determine the mRNA expression of *Il17a* and *Il1b* in the colonic epithelium from AOM-DSS-treated *Zfp90*^fl/fl^ and *Zfp90*^ΔIEC^ mice (n = 5 per group). **j** GSEA was used to identify the differential gene profiles between the colonic epithelium from AOM-DSS-treated *Zfp90*^fl/fl^ and *Zfp90*^ΔIEC^ mice. **k** Representative immunohistochemical staining for LY6G and F4/80 in colon segments from AOM-DSS-treated *Zfp90*^fl/fl^ and *Zfp90*^ΔIEC^ mice. Data with error bars represent the mean ± SD. Each panel is a representative experiment of at least three independent biological replicates. Mann–Whitney U test (a, h), nonpaired two-tailed t-test (b, d, i).

The barrier function was also evaluated in the acute colitis model by testing FITC-dextran and LPS level in the serum of the *Zfp90*^fl/fl^ and *Zfp90*^ΔIEC^ mice (Supplementary SFigure 2a, b). Consistent with the CAC model, the *Zfp90*^fl/fl^ mice showed more FITC-dextran leakage and increased LPS translocation than the *Zfp90*^ΔIEC^ mice under DSS treatment. Moreover, the level of *Zo-1* was decreased in colon tissue of the DSS-treated *Zfp90*^fl/fl^ mice as compared with that in the *Zfp90*^ΔIEC^ mice (Supplementary SFigure 2c).

The gut barriers in the AOM-DSS-treated *Zfp90*^fl/fl^ and *Zfp90*^ΔIEC^ mice showed different stabilities; therefore, we decided to determine the extent of colon inflammation in the two groups. RNA sequencing (RNA-seq) of the colon epithelium was conducted and the transcriptomes of the two groups were compared. Principal component analysis (PCA) showed that the transcripts were separately clustered according to genotype (Supplementary SFigure 2d). Kyoto Encyclopedia of Genes and Genomes (KEGG) analysis revealed that the genes differentially expressed between the *Zfp90*^fl/fl^ and *Zfp90*^ΔIEC^ mice could be categorized into 27 pathways related to the immune system, signal transduction, and cancers (Supplementary SFigure 2e), such as the “Cytokine-cytokine receptor interaction”, “Inflammatory bowel disease (IBD)” and “NF-κB signaling pathway”. Gene expression in the “Cytokine-cytokine receptor interaction” pathway clearly showed milder colonic inflammation in the *Zfp90*^ΔIEC^ mice compared with that in the *Zfp90*^fl/fl^ mice (Figure 2g). The decreased expression of *Il17a* (encoding interleukin 17A (IL17A)) and *Il1b* (encoding interleukin 1 beta (IL1β)) in the colon epithelium of AOM-DSS-treated *Zfp90*^ΔIEC^ mice were also confirmed using real-time PCR (Figure 2(h, i)). Gene set enrichment analysis (GSEA) revealed that the gene sets related to GO_NEUTROPHIL_MIGRATION were enriched in the *Zfp90*^fl/fl^ mice (Figure 2j). Accordingly, our immunohistochemistry analysis of lymphocyte antigen 6 complex locus G6D (LY6G) showed higher abundance of neutrophil infiltration in colon segments from the *Zfp90*^fl/fl^ mice (Figure 2k, Supplementary SFigure 2f). Additionally, increased infiltration of macrophages was also detected in lesions from the colons of the *Zfp90*^fl/fl^ mice using F4/80 (also known as EGF module-containing mucin-like receptor) staining (Figure 2k, Supplementary SFigure 2g).

Taken together, these data revealed that the *Zfp90*^ΔIEC^ mice possessed a relatively integral gut barrier upon AOM-DSS treatment, compared with the *Zfp90^f^*^l/fl^ mice. A better gut barrier function reduced the translocation of small molecules such as LPS and even gut bacteria themselves, thus leading to an overall milder inflammatory response.

### *The gut microbiota is indispensable for the antitumor phenotype in* Zfp90*^ΔIEC^ mice*

Recent research indicated that gut microbiota is involved in the development of CAC.^6^ Therefore, we tried to determine whether the gut microbiota contributes to the discrepancy between *Zfp90*^fl/fl^ and *Zfp90*^ΔIEC^ mice.

**Figure 3.**
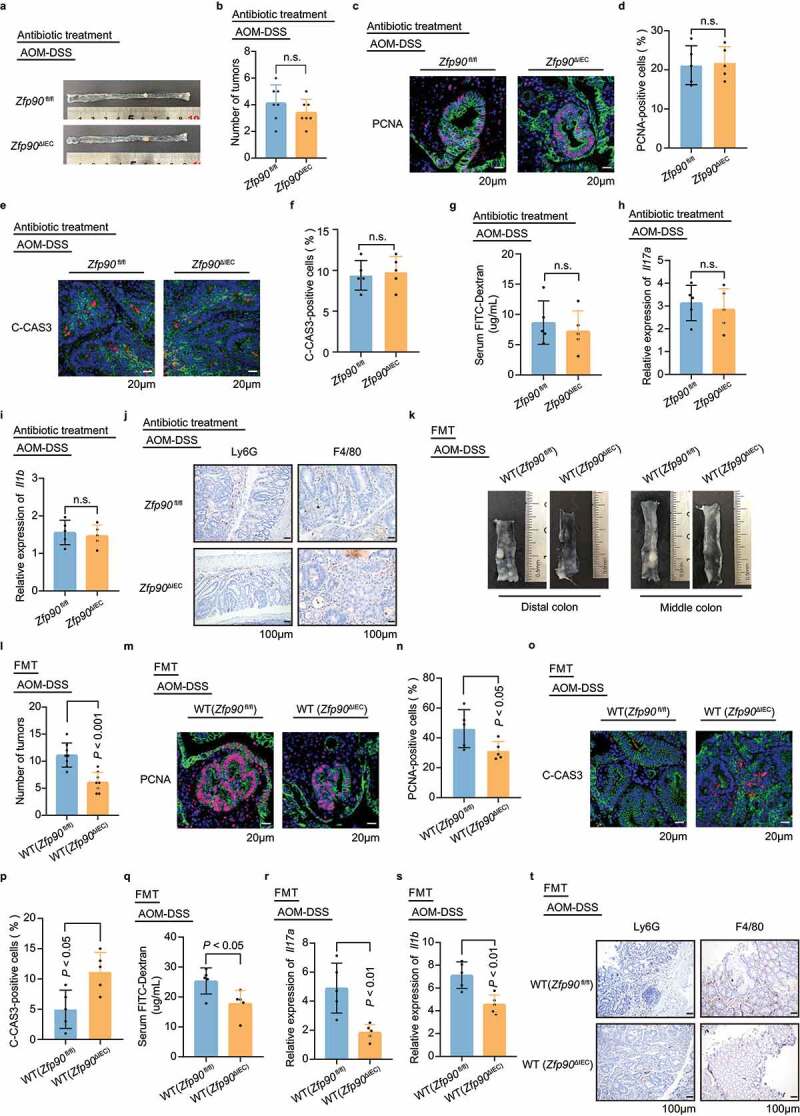
**The gut microbiota is indispensable for the antitumor phenotype in *Zfp90*^ΔIEC^ mice. a** Representative images of colons in antibiotic-treated *Zfp90*^fl/fl^ and *Zfp90*^ΔIEC^ mice after AOM-DSS treatment. **b** Tumor numbers in antibiotic-treated *Zfp90*^fl/fl^ and *Zfp90*^ΔIEC^ mice (n = 7 per group). **c** Representative immunofluorescence staining of the PCNA protein in tumor tissues from antibiotic-treated *Zfp90*^fl/fl^ and *Zfp90*^ΔIEC^ mice after AOM-DSS treatment. Sections were stained with DAPI (blue), CDH1 (green) and PCNA (red). **d** The percentage of PCNA-positive tumor cells was quantified (n = 5 per group). **e** Representative immunofluorescence staining of C-CAS3 protein in tumor tissues from antibiotic-treated *Zfp90*^fl/fl^ and *Zfp90*^ΔIEC^ mice after AOM-DSS treatment. Sections were stained with DAPI (blue), CDH1 (green) and C-CAS3 (red). **f** The percentage of C-CAS3-positive tumor cells was quantified (n = 5 per group). **g** Intestinal permeability assessed by FITC-dextran in antibiotic-treated *Zfp90*^fl/fl^ and *Zfp90*^ΔIEC^ mice (n = 5 per group). **h, i** Real-time PCR was performed to determine the mRNA expression of *Il17a* and *Il1b* in the colonic epithelium from antibiotic-treated *Zfp90*^fl/fl^ and *Zfp90*^ΔIEC^ mice (n = 5 per group). **j** Representative immunohistochemical staining for LY6G and F4/80 in colon segments from antibiotic-treated *Zfp90*^fl/fl^ and *Zfp90*^ΔIEC^ mice with AOM-DSS treatment. **k-t** Gavage of WT mice with feces from *Zfp90*^fl/fl^ and *Zfp90*^ΔIEC^ mice (n = 5–8 per group). Representative images of distal and middle colons (k), number of tumors (l), representative immunofluorescence staining of PCNA (m), percentage of PCNA-positive tumor cells (n), representative immunofluorescence staining of C-CAS3 (o), percentage of C-CAS3-positive tumor cells (p), the serum FITC-Dextran level (q), mRNA expression of *Il17a* and *Il1b* in the colonic epithelium (r, s), and representative immunohistochemical staining for LY6G and F4/80 (t) from WT (*Zfp90*^fl/fl^ mice) and WT (*Zfp90*^ΔIEC^ mice) under AOM-DSS treatment. Sections were stained with DAPI (blue), CDH1 (green) and PCNA or C-CAS3 (red). Data with error bars represent the mean ± SD. Each panel is a representative experiment of at least three independent biological replicates. Nonpaired two-tailed t-test (b, d, f, g, h, i, l, n, p, r, s) and the Mann–Whitney U test (q) were used

To confirm the role of the gut microbiota in the tumorigenesis of *Zfp90*^ΔIEC^ and *Zfp90*^fl/fl^ mice, the gut microbiota was depleted in *Zfp90*^fl/fl^ and *Zfp90*^ΔIEC^ mice using an antibiotic cocktail for 2 weeks before AOM-DSS treatment. The efficacy of the microbiota depletion was confirmed by quantification of fecal 16S rRNA gene levels (Supplementary SFigure 3a). Depletion of the microbiota eliminated the differences in colon tumor number between the two groups (Figure 3(a, b)). Furthermore, cell proliferation and apoptosis, as shown by PCNA and C-CAS3 levels, were similar between the two groups after elimination of the microbiota (Figure 3(c-f)). We then evaluated the gut permeability in *Zfp90*^fl/fl^ and *Zfp90*^ΔIEC^ mice and found no differences in FITC-dextran leakage between the two groups, indicating a similar decline in gut barrier function after AOM-DSS treatment (Figure 3g). Accordingly, real-time PCR showed that the epithelial expression of *Il17a* and *Il1b* became similar between the *Zfp90*^fl/fl^ and *Zfp90*^ΔIEC^ mice after antibiotic treatment (Figure 3(h, i)). Immunohistochemistry image of LY6G and F4/80 showed similar abundance of neutrophil and macrophage infiltration in colon segments from Zfp90^fl/fl^ and *Zfp90*^ΔIEC^ mice (Figure 3j, Supplementary SFigure 3b).

We then sought to explore whether fecal microbiota transfer (FMT) could confer the antitumor ability of *Zfp90*^ΔIEC^ mice to wild-type (WT) C57BL/6 J mice. Stools from mono-housed tumor-bearing *Zfp90*^fl/fl^ and *Zfp90*^ΔIEC^ mice were gavaged to antibiotic-treated WT mice in SPF background (Supplementary SFigure 3c). Gavage of antibiotic-treated mice with feces from the *Zfp90*^ΔIEC^ mice led to a reduced number of colon tumors compared with those receiving feces from the *Zfp90*^fl/fl^ mice under AOM-DSS treatment (Figure 3(k, l). Mice transplanted with fecal pellets from the *Zfp90*^ΔIEC^ mice (WT (*Zfp90*^ΔIEC^ mice)) also had lower proliferative activity and increased apoptosis compared with mice transplanted with fecal pellets from the *Zfp90*^fl/fl^ mice (WT (*Zfp90*^fl/fl^ mice)) (Figure 3(m-p)). Next, gut permeability was assessed between the two groups and a lower level of serum FITC-dextran was found in the WT (*Zfp90*^ΔIEC^) mice (Figure 3q). Real-time PCR showed lower expression of *Il17a* and *Il1b* in the WT (*Zfp90*^ΔIEC^) mice compared with that in WT (*Zfp90*^fl/fl^) mice (Figure 3(r, s)). Immunohistochemistry of LY6G and F4/80 showed higher levels of neutrophil and macrophage infiltration in colon segments from the WT (*Zfp90*^fl/fl^) mice than in those from the WT (*Zfp90*^ΔIEC^) mice (Figure 3t, Supplementary SFigure 3d).

We next investigated whether the gut microbiota also mediated the phenotypic variation in acute colitis model. Gut microbiota depletion was conducted for 2 weeks before the onset of DSS treatment. The severity of colitis was comparable between *Zfp90*^fl/fl^ and *Zfp90*^ΔIEC^ mice, as indicated by similar weight loss (Supplementary SFigure 3e) and colon length (Supplementary SFigure 3f). There were no differences in FITC-dextran leakage between the *Zfp90*^fl/fl^ and *Zfp90*^ΔIEC^ mice after 7 days of DSS treatment (Supplementary SFigure 3g). Furthermore, no significant differences in *Zo-1* levels were detected between the colon tissues of the *Zfp90*^fl/fl^ and *Zfp90*^ΔIEC^ mice (Supplementary SFigure 3h).

Taken together, these results indicated that antitumor phenotype in the *Zfp90*^ΔIEC^ mice is dependent on the gut microbiota and recipients transplanted with fecal microbiota from *Zfp90*^ΔIEC^ mice could phenocopy their donors.

### *A* Prevotella copri*-defined microbiota underlies the mechanism of the deleterious effect of oncogene* Zfp90

**Figure 4.**
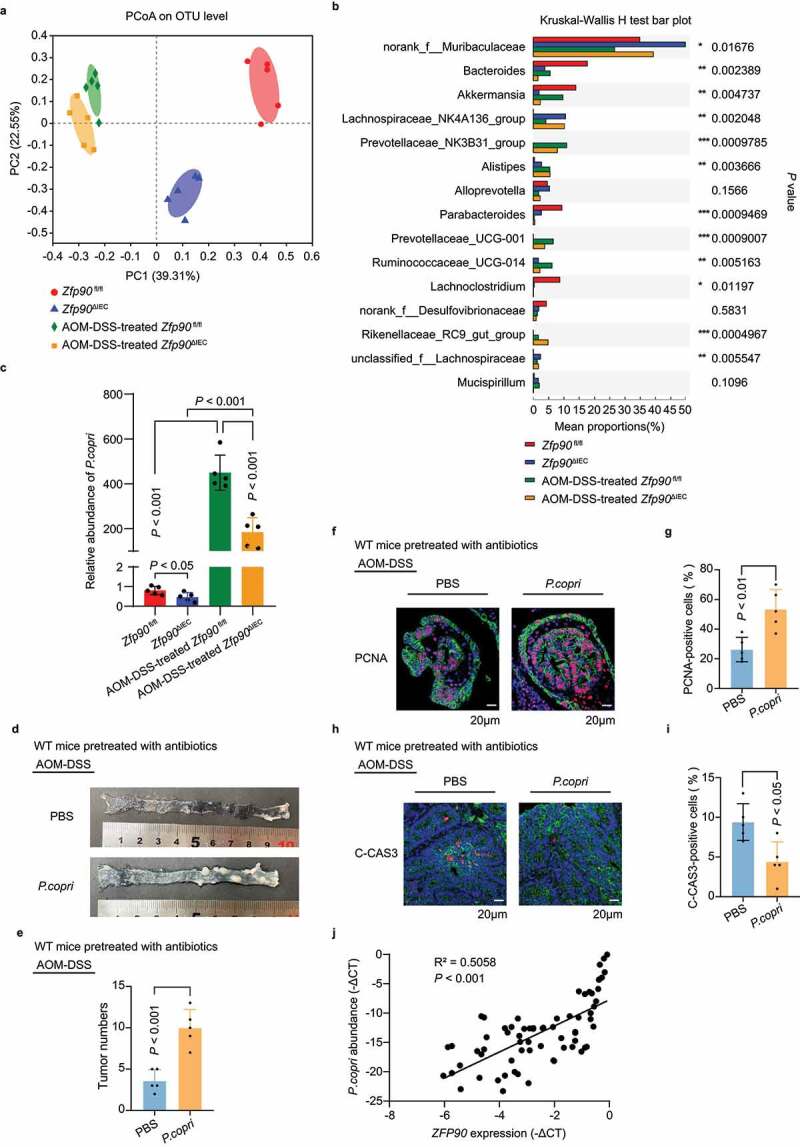
**The gut microbiota is altered by intestine-specific deletion of oncogene *Zfp90*. a** Principal component analysis plot based on bacterial 16S ribosomal DNA gene sequences of the fecal content from *Zfp90*^fl/fl^ and *Zfp90*^ΔIEC^ mice before and after AOM-DSS-treatment (n = 5 per group). **b** Differential abundances of bacteria in the fecal content of *Zfp90*^fl/fl^ and *Zfp90*^ΔIEC^ mice before and after AOM-DSS-treatment. **c** Real-time PCR was performed to determine the abundance of *P. copri* in the fecal content of *Zfp90*^fl/fl^ and *Zfp90*^ΔIEC^ mice before and after AOM-DSS-treatment. **d** Representative images of colons in PBS- and *P. copri*-treated WT mice after AOM-DSS treatment. **e** Tumor numbers in PBS- and *P. copri*-treated WT mice after AOM-DSS treatment. **f** Representative immunofluorescence staining of the PCNA protein in tumor tissues from PBS- and *P. copri*-treated WT mice after AOM-DSS treatment. Sections were stained with DAPI (blue), CDH1 (green) and PCNA (red). **g** The percentage of PCNA-positive tumor cells was quantified. **h** Representative immunofluorescence staining of C-CAS3 protein in tumor tissues from PBS- and *P. copri*-treated WT mice after AOM-DSS treatment. Sections were stained with DAPI (blue), CDH1 (green) and C-CAS3 (red). **i** The percentage of C-CAS3-positive tumor cells was quantified. **j** The correlation between *P. copri* abundance and *ZFP90* expression in specimens from patients with CRC. Statistical analysis was performed using nonpaired two-tailed t-test (e, g, i), one-way ANOVA (c), and Wilcoxon’s rank-sum test (j)

To explore the fecal bacterial composition in the *Zfp90*^fl/fl^ and *Zfp90*^ΔIEC^ mice, we performed 16S ribosomal DNA sequencing. PCA based on operational taxonomic unit (OTU) levels showed distinct gut microbiota compositions in the two groups under both naïve and AOM-DSS-treated conditions (Figure 4a **and** Supplementary SFigure 4a). Depletion of *Zfp90* resulted in an increase in the community diversity and richness, as indicated by the Shannon and Sobs indices, respectively (Supplementary SFigure 4b, c), suggesting that the gut microbiota of the *Zfp90*^ΔIEC^ mice had more species variation than that of the *Zfp90*^fl/fl^ mice. KEGG analysis indicated that “lipopolysaccharide (LPS) biosynthesis proteins” was one of the key metabolic pathways downregulated in the *Zfp90*^ΔIEC^ mice (Supplementary SFigure 4d). The different bacterial compositions between groups were further analyzed at the genus level. The abundance of *Prevotellaceae_NK3B31_group* and *Prevotellaceae_UCG-001* was low in both the *Zfp90*^fl/fl^ and *Zfp90*^ΔIEC^ mice before AOM-DSS treatment, while the abundance increased significantly at the end of the treatment, especially in the *Zfp90*^fl/fl^ mice (Figure 4b). Therefore, we speculated that the family *Prevotellaceae* could contribute to the AOM-DSS-induced CAC and might drive the different phenotypes of the two groups under these specific conditions. The family *Prevotellaceae* is composed of four genera, of which the genus *Prevotella* is known for its role in chronic inflammatory disease.^36^ The *Prevotella* genus encompasses more than 40 different culturable species, of which the *Prevotella copri* (*P. copri*) is rich among gut-commensal bacteria. Recent studies correlated higher abundance of *P. copri* with intestinal infection,^37^ insulin resistance,^38^ higher incidence of colon cancer,^39^ enhancement of rheumatoid arthritis,^40^ and metabolic syndrome.^41^ Therefore, we hypothesized that *P. copri* might be the driver microbe for CAC development in this context. To verify this hypothesis, real-time PCR was conducted in fecal pellets from untreated or AOM-DSS-treated *Zfp90*^fl/fl^ and *Zfp90*^ΔIEC^ mice. The abundance of *P. copri* in the fecal pellets from both mice increased significantly after AOM-DSS treatment and the abundance was markedly higher in the *Zfp90*^fl/fl^ mice at the end of AOM-DSS treatment (Figure 4c).

To confirm the effect of *P. copri* on AOM-DSS-induced colorectal tumorigenesis, WT mice pretreated with antibiotics for 2 weeks were gavaged with 10^8^ colony-forming units (cfu) of *P. copri* or phosphate-buffered saline (PBS) every other day during AOM-DSS treatment (Supplementary SFigure 4e). Eighty days after the induction of tumorigenesis, increased tumor number was observed in the mice colonized with *P. copri* (Figure 4(d, e)). Moreover, the mice colonized with *P. copri* exhibited higher proliferative activity and less apoptosis than the mice gavaged with PBS (Figure 4(f-i)). We then explored the role of *P. copri* in the acute colitis model. After 2 weeks of antibiotic treatment, the mice were first gavaged with *P. copri* for 1 week and then given DSS for 7 days, with the gavage of *P. copri* continuing throughout the experiment. More severe colitis was observed in the mice colonized with *P. copri* (Supplementary SFigure 4f, g). The gut barrier dysfunction was more severe in the mice colonized with *P. copri*, as indicated by more FITC-dextran leakage, more LPS translocation, and lower levels of *Zo-1* (Supplementary SFigure 4h-j).

We then sought to determine the correlation between the expression of *ZFP90* and the abundance of *P. copri* in clinical patients. There was a lack of CAC specimens; therefore, we verified the relationship in patients with CRC, because chronic inflammation is frequently observed in this population.^16^ Remarkably, a positive correlation was detected between the abundance of *P. copri* and the expression of *ZFP90* in 81 patients with CRC (Figure 4j).

Taken together, our results indicated that *P. copri* plays an important role in the development of CAC, and that the characteristics caused by a *P. copri-*defined microbiota, including increased LPS biosynthesis and a compromised gut barrier function, might partly account for the susceptibility of *Zfp90*^fl/fl^ mice to colitis and AOM-DSS-induced CAC.

### *Decreased TLR4-dependent PI3K/AKT/NF-κB signaling in the colon tissues of* Zfp90*^ΔIEC^ mice*

Researchers have found that antigen presenting cells (APCs) in the *Prevotella*-rich mucosa showed a profile similar to those activated by LPS.^42^ Considering the increased abundance of *P. copri* and the enrichment of the “LPS biosynthesis proteins” pathway in *Zfp90*^fl/fl^ mice, we speculated that a *P. copri-*defined microbiota might have a more potent ability to produce LPS. Accompanied by a compromised gut barrier, the elevated production of LPS might then translocate and play a vital role in the development of CAC. Previous studies demonstrated that LPS could bind with Toll-like receptor 4 (TLR4) at the surface of intestinal cells and triggered signal transductions and activated the expression of genes encoding inflammatory effectors, such as nuclear factor κB (NF-κB).^43,44^

**Figure 5.**
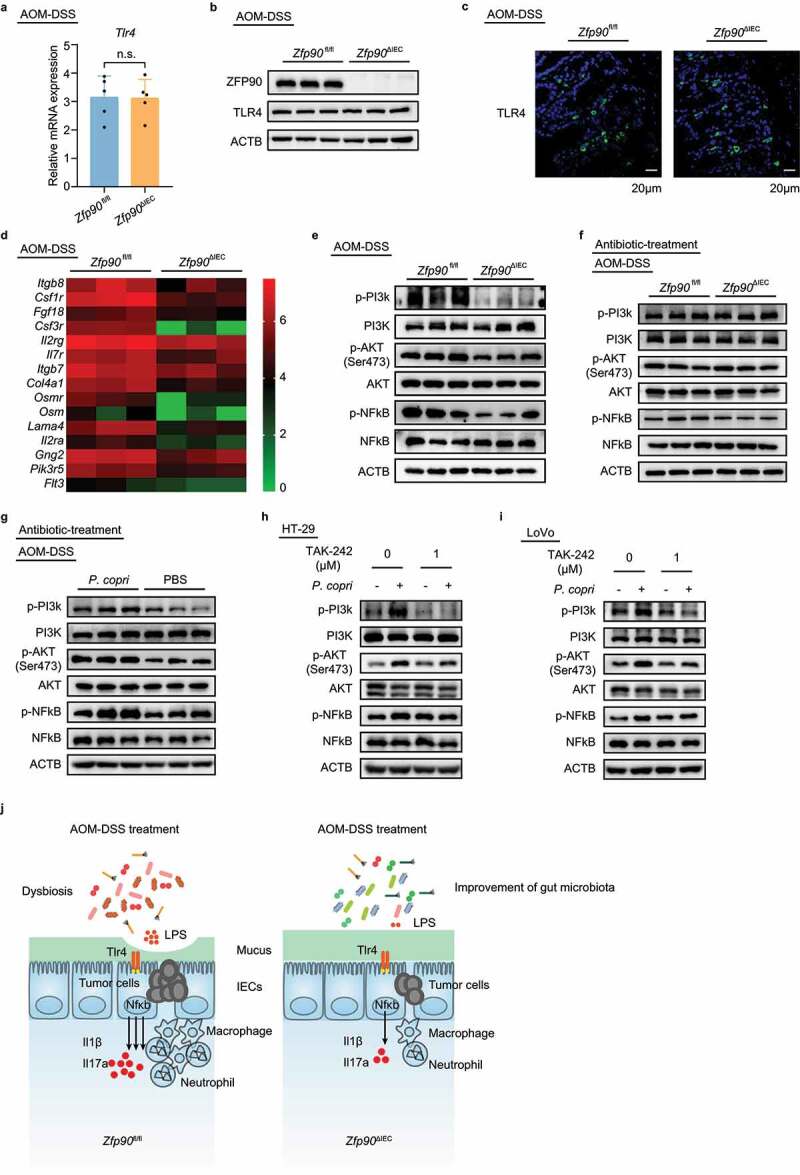
Decreased TLR4-dependent PI3K/AKT/NF-κB signaling in colon tissue of *Zfp90*^ΔIEC^ mice

Based on these results, we first detected the expression of TLR4 in the *Zfp90*^ΔIEC^ and *Zfp90*^fl/fl^ mice using real-time PCR, western blot, and immunofluorescence. However, the expression of TLR4 in the colon tissue was not significantly different between mono-housed *Zfp90*^ΔIEC^ and *Zfp90*^fl/fl^ mice during AOM-DSS treatment (Figure 5(a-c)). These data revealed that deletion of *Zfp90* in the colon epithelium did not change the expression of TLR4.

**a** Real-time PCR was performed to determine the mRNA expression of *Tlr4* in AOM-DSS-treated *Zfp90*^fl/fl^ and *Zfp90*^ΔIEC^ mice (n = 5 per group). **b** Representative data showing protein level of TLR4 in AOM-DSS-treated *Zfp90*^fl/fl^ and *Zfp90*^ΔIEC^ mice. **c** Representative immunofluorescence staining of the TLR4 protein in tumor tissues from AOM-DSS-treated *Zfp90*^fl/fl^ and *Zfp90*^ΔIEC^ mice. Sections were stained with DAPI (blue) and TLR4 (green). **d** Heatmap representing log_2_-normalized count of the PI3K-AKT pathway expression on the colonic epithelium from *Zfp90*^fl/fl^ and *Zfp90*^ΔIEC^ mice (n = 3 per group). **e** Representative data showing the activity of the PI3K-AKT-NFκB pathway in AOM-DSS-treated *Zfp90*^fl/fl^ and *Zfp90*^ΔIEC^ mice. **f** Representative data showing the activity of the PI3K-AKT-NFκB pathway in antibiotic-treated *Zfp90*^fl/fl^ and *Zfp90*^ΔIEC^ mice after AOM-DSS treatment. **g** Representative data showing the activity of the PI3K-AKT-NFκB pathway in PBS- and *P. copri*-treated WT mice after AOM-DSS treatment. **h, i** HT-29 (**h**) and LoVo (**i**) cell lines were pretreated with TLR4 inhibitor (TAK-242) 30 minutes before co-culturing with *P. copri*. Representative data showing the effect of TLR4 inhibitor on the activation of the PI3K-AKT-NFκB pathway. **j** Model for the *P. copri*-defined microbiota-mediated colorectal tumorigenesis. Data with error bars represent the mean ± SD. Each panel is a representative experiment of at least three independent biological replicates. Statistical analysis was performed using nonpaired two-tailed t-test.

To determine whether the deletion of *Zfp90* in the colon epithelium had an impact on the activation of TLR4, we assessed the activity of its downstream signaling pathway. It has been established that phosphatidylinositol-4,5-bisphosphate 3-kinase (PI3K) -protein kinase B (AKT)-NF-κB is one of primary signaling transduction pathways following the activation of TLR4.^45,46^ In accordance with this, our RNA-seq results revealed that the “PI3K-Akt signaling pathway” was downregulated in the *Zfp90*^ΔIEC^ mice (Supplementary SFigure 2e, Figure 5d). The inhibitory function of *Zfp90* deletion on the activity of the PI3K-Akt signaling pathway was confirmed by western blotting (Figure 5e). Whereas the mRNA expression levels of *Pi3k, Akt*, and *Nfkb* showed no differences between the *Zfp90*^fl/fl^ and *Zfp90*^ΔIEC^ mice (Supplementary Figure 5a-c). To further verify that the gut microbiota mediated the activation of PI3K/AKT/NF-κB signaling, we detected the activity of this pathway in antibiotic-treated *Zfp90*^fl/fl^ and *Zfp90*^ΔIEC^ mice. At the end of AOM-DSS treatment, no significant differences were found in the activity of the PI3K-AKT signaling pathway between antibiotic-treated *Zfp90*^fl/fl^ and *Zfp90*^ΔIEC^ mice (Figure 5f). Moreover, stronger activation of PI3K/AKT/NF-κB signaling was observed in antibiotic-treated WT mice colonized with *P. copri* (Figure 5g).

To further verify that the activation of PI3K/AKT/NF-κB signaling was TLR4-dependent, HT-29 cells and LoVo cells were first treated with a TLR4 inhibitor (TAK-242) and then co-cultured with *P. copri*. Western blotting showed that *P. copri*-mediated PI3K-AKT-NF-κB activation was largely abrogated by the TLR4 inhibitor (Figure 5(h, i)), corroborating the role of TLR4 in this process.

Taken together, these data showed that a *P. copri*-defined microbiota might participate in the development of CAC by activating TLR4 and the PI3K-AKT-NF-κB signaling pathway (Figure 5j).

## Discussion

ZFP90 is a zinc-finger protein containing a KRAB box. Dysregulation of ZFP90 has been proven to be associated with several diseases including obesity,^47^ cardiac dysfunction,^48^ and mental retardation.^49^ In addition, we previously showed that *ZFP90* is a target gene of CRC susceptibility locus 16q22.1^32^. Using an AOM-induced sporadic CRC mouse model and clinical specimens from patients with CRC, we validated its oncogenic role in the CRC. However, the underlying mechanisms of ZFP90 in the development of CAC warrant further investigation.

In this study, through overlapping findings from GWAS and the GEO database, we found that *ZFP90* might be a key gene that regulates the pathogenesis of CAC. We observed that conditional knock-out of *Zfp90* in IECs could protect mice from AOM-DSS-induced CAC.

Intestinal barrier breach and subsequent inflammation have been proven to contribute to chronic inflammatory and malignant colorectal diseases;^50^ therefore, we compared the gut permeability in the AOM-DSS-treated Zfp90^fl/fl^ mice with that in the AOM-DSS-treated *Zfp90*^ΔIEC^ mice. As expected, the *Zfp90*^fl/fl^ mice displayed significantly higher intestinal permeability. Furthermore, our RNA-seq and PCR results confirmed higher inflammatory responses in the *Zfp90*^fl/fl^ mice after AOM-DSS treatment. Taken together, our results suggested that the increased gut permeability in the *Zfp90*^fl/fl^ mice might contribute to the exaggerated inflammation and subsequent AOM-DSS-induced carcinogenesis.

Accumulating evidence demonstrates that the human gut microbiota is involved in various pathological conditions, including CRC. We therefore investigated whether the gut microbiota actively mediates the protective role of *Zfp90* deletion in AOM-DSS-induced CAC. We treated *Zfp90*^fl/fl^ and *Zfp90*^ΔIEC^ mice with antibiotics before the initiation of AOM-DSS treatment. After depletion of the microbiota, we failed to find differences between the two groups in terms of tumor development, gut permeability, and inflammation. We then employed FMT, and found that mice transplanted with the fecal microbiota from *Zfp90*^fl/fl^ and *Zfp90*^ΔIEC^ mice phenocopied their donors in terms of tumor development, gut permeability, and inflammation. Furthermore, our high-throughput sequencing results indicated that the microbiota compositions were different between AOM-DSS-treated *Zfp90*^ΔIEC^ and Zfp90^fl/fl^ mice. Specifically, we detected a higher abundance of *P. copri* in the *Zfp90*^fl/fl^ mice. It has been reported that a *P. copri*-defined microbiota was associated with exacerbated colitis in mice.^37,51^ Accordingly, our functional analysis demonstrated that “lipopolysaccharide (LPS) biosynthesis proteins” was one of the key metabolic pathways that was upregulated in the microbiota of the *Zfp90*^fl/fl^ mice. Considering that the gut barrier was greatly compromised in the Zfp90^fl/fl^ mice during AOM-DSS treatment, we therefore speculated that the increased LPS production from the intestinal flora could subsequently translocate and induce inflammation by activating TLR4^5^. As such, a *P. copri*-defined microbiota would exacerbate inflammation and contribute to the subsequent development of AOM-DSS-induced CAC.

To further corroborate the enhanced activation of TLR4 by LPS, the expression of TLR4 and activity of its downstream signaling pathway were assessed. Although the expression of TLR4 was not significantly different between *Zfp90*^fl/fl^ and *Zfp90*^ΔIEC^ mice, PI3K-AKT-NF-κB signaling pathway activation was strongly decreased in the *Zfp90*^ΔIEC^ mice. PI3K-AKT-NF-κB is one of primary signaling transduction pathways responsible for the synthesis of pro-inflammatory mediators following the activation of TLR4.^52,53^ Our *in vitro* experiments further demonstrated that pretreatment with a TLR4 inhibitor could greatly reduce *P. copri-*induced activation of PI3K-AKT-NF-κB signaling. Thus, these results validated that TLR4 activation was increased by the *P. copri*-defined microbiota, which then contributed to the development of inflammatory and malignant pathologies.

In conclusion, our work demonstrated the oncogenic role of *Zfp90* in AOM-DSS-induced CAC and this relationship is, at least partly, mediated by the gut microbiota. The *P. copri*-defined microbiota in *Zfp90*^fl/fl^ mice promotes the production and translocation of LPS upon a compromised gut barrier, which contributes to the subsequent inflammatory and carcinogenic processes by activating the TLR4-dependent PI3K-AKT-NF-κB signaling pathway. This study highlighted that manipulation of the gut microbiota might represent a potential strategy to prevent and treat CAC.

## Methods

### Study population and clinical specimens

Patients with CRC were recruited from Renji Hospital affiliated to Shanghai Jiao Tong University School of Medicine. All the patients with CRC were of Han Chinese ethnicity. Written informed consents were obtained from all participants in this study. All the research was carried out in accordance with the provisions of the Helsinki Declaration of 1975. There were 81 snap frozen, colorectal cancerous tissues. DNA and RNA were extracted from the snap frozen tissues using an AllPrep DNA/RNA Mini Kit (QIAGEN, Hilden, Germany) under the manufacturer’s guidelines to detect *ZFP90* expression and *P. copri* abundance.

### *Generation of* Zfp90*^ΔIEC^ Mice*

*Zfp90*^ΔIEC^ mice have been described previously.^32^ Briefly, to generate the *Zfp90*^fl/+,Villin-cre/+^ mice, exon 4 and the 3′ UTR were flanked by loxP sites using C57BL/6 J ES cells with a 2 kb fragment upstream and downstream as the 5′ and 3′ arms. Then, the *Zfp90*^fl/+^mice were crossed to C57BL/6 J Villin-cre mice to generate *Zfp90*^fl/+,Villin-cre/+^ mice. *Zfp90*^fl/+,Villin-cre/+^ mice were each intercrossed to generate homozygous knockout mice (*Zfp90*^ΔIEC^) and littermates (*Zfp90*^fl/fl^). Mice were genotyped via PCR and Sanger sequencing using genomic DNA obtained from mouse tails.

All mice were bred in an SPF environment. Mouse experiments were conducted in accordance with the National Institutes of Health Guidelines for the Care and Use of Laboratory Animals. The study procedures were approved by the Institutional Animal Care and Use Committee of Renji Hospital, Shanghai Jiao Tong University School of Medicine.

### Mouse model

To construct the CAC model,^54^ *Zfp90*^fl/fl^ and *Zfp90*^ΔIEC^ mice at 8 weeks old were injected intraperitoneally with AOM (10 mg/kg, Sigma-Aldrich, St. Louis, MO, USA). After 5 days, 2.5% DSS was added to the drinking water for 7 consecutive days, followed by 14 days of regular water. Three cycles of DSS treatment were used. The mice were sacrificed on day 80 after the induction of tumorigenesis. All colon tissues, tumor tissues, feces, serum, and spleens were collected on day 80. To deplete the gut microbiota, drinking water supplemented with an antibiotic cocktail (0.5 g/L of ampicillin, neomycin, and metronidazole and 0.25 g/L of vancomycin) was given to the mice for 2 weeks before AOM-DSS treatment and the procedure was repeated once in the middle of the experiment. For the gut microbiota transfer experiment, fresh stool samples from tumor-bearing *Zfp90*^fl/fl^ and *Zfp90*^ΔIEC^ mice were collected, suspended in 30% glycerin/PBS, aliquoted, and stored at – 80 °C. Fecal microbiota was then gavaged into the antibiotic-treated mice three times a week. Colons were removed and were flushed with cold PBS after the mice were sacrificed. The colon length, tumor number, and tumor size were measured for each mouse. The colons of different experimental batches were used for histopathological analysis or epithelium cell isolation. Spleens were also separated from each mouse.

### Isolation of colonic epithelium cells

Colonic epithelium cells were isolated as described previously, with some modifications.^55^ Colons were collected from mice and cleared by holding with forceps and flushing with a syringe filled with ice-cold PBS. Next, the colons were opened longitudinally and cut into 1–2 cm pieces, followed by several PBS washes. The pieces were then incubated with 2.5 mM ethylenediaminetetraacetic acid (EDTA) and 1 mM Dithiothreitol (DTT) in Hank’s balanced salt solution (HBSS) for 20 min at 37 °C under slow rotation. Supernatants were then passed through a 100 μm cell strainer. Colonic epithelium cells were collected by centrifugation at 800 × *g* and were used for the following analysis.

### Immunohistochemistry (IHC)

For immunohistochemistry, 4-μm-thick paraffin colon tissue slides were subjected to heat-induced antigen retrieval, followed by peroxidase blocking treatment to remove endogenous hydrogen peroxide. After blocking with 10% sheep serum in PBS, the slides were incubated with anti-LY6G (Santa Cruz Biotechnology, Santa Cruz, CA, USA) and anti-F4/80 (Santa Cruz Biotechnology) antibodies in a humidified chamber at 4°C overnight. Then, the slides were incubated with horseradish peroxidase (HRP)-labeled secondary antibodies at room temperature for 30 minutes and then stained with 3,3’-Diaminobenzidine (DAB) substrate solution.

### Immunofluorescence staining and confocal microscopy

For immunofluorescence, 4-μm-thick paraffin colon tissue slides were subjected to heat-induced antigen retrieval, followed by sodium borohydride treatment to remove tissue autofluorescence. After blocking with 5% bovine serum albumin in PBS, the slides were incubated with anti-C-CAS3 (Cell Signaling Technology (CST), Danvers, MA, USA) and anti-PCNA (Santa Cruz Biotechnology) antibodies in a humidified chamber at 4°C overnight. Then, the slides were incubated with fluorophore-labeled secondary antibodies at room temperature for 1 hour. After rinsing with PBS, the slides were mounted using antifade mounting medium with 4′,6-diamidino-2-phenylindole (DAPI), and the fluorescence was detected under a laser-scanning confocal microscope.

### *Fluorescence* in situ *hybridization (FISH)*

FISH was performed as described previously.^56^ Briefly, colon tissues with fecal pellets were fixed in methanol-carnoy fixative (60% methanol, 30% chloroform, and 10% glacial acetic acid). After fixation, tissues were washed with the following solutions: twice in methanol, twice in ethanol, and twice in xylene, and then embedded in paraffin. Paraffin sections of 6 μm were subjected to mucin 2 staining with fluorescein-labeled Anti-*Ulex Europaeus*-I (UEA-I) lectin, and bacteria were detected using the universal bacteria FISH probe, 5′-Cy3-labeled EUB338I. After rinsing with washing buffer, the slides were mounted with DAPI and imaged under a laser-scanning confocal microscope.

### FITC–dextran intestinal permeability assay

Colonic permeability was assessed by oral gavage of 4 kDa FITC-labeled dextran (Sigma-Aldrich). Mice were fasted for 3 hours before the assay and then administered with 150 μL FITC-dextran (80 mg/mL). After 4 hours, 100 μL of blood was collected and centrifuged at 10000 × *g* for 10 minutes to acquire serum. The serum was diluted 1:4 in water and 100 μL/well was added to 96-well plates. The FITC-labeled dextran levels were then measured by fluorometry with 485 excitation/528 emission.

### Analysis of LPS levels in serum

The serum LPS levels were quantified using a simple and sensitive amebocyte lysate assay (Pierce Chromogenic Endotoxin Quant Kit, Pierce Biotechnology, Rockford, IL, USA). The experiment was conducted according to the manufacturer’s instructions. Briefly, serum samples were diluted 50-fold and heat-shocked at 70 °C for 15 minutes. Then, the Amebocyte Lysate Reagent, the pre-warmed Chromogenic Substrate Solution, and the Stop Solution were added sequentially added to the serum sample. The optical density (OD) was read at 405 nm immediately after assay completion.

### Cell lines

Human CRC cell lines, HT-29 and LoVo were used in the study. All CRC cell lines were cultured according to ATCC culture methods. HT-29 was cultured in McCoy’s 5a Medium (GIBCO, Carlsbad, CA) supplemented with 10% fetal bovine serum (FBS) (GIBCO, Carlsbad, CA). LoVo was cultured in F-12 K Medium (GIBCO, Carlsbad, CA) supplemented with 10% FBS. These two cell lines were cultured at 37°C with 5% CO2.

### Western Blot and Chemical Reagents

Western blot was performed using standard protocols. Cell extracts were collected and quantified using BCA Protein Assay Kit (Thermo Scientific, Rockford, U.S.A.). 40 μg of protein was electrophoresed through 7.5% SDS polyacrylamide gels and were then transferred to PVDF membranes (Bio-Rad, Hercules, CA). The membranes were blocked in 5% BSA for one hour and then incubated with primary antibodies at 4°C overnight. Secondary antibodies were labeled with HRP (KangChen, China) and the signals were detected using Pierce ECL Western Blotting Substrate (Thermo Scientific, Rockford, U.S.A.) by ChemiDoc Imaging System (BIO-RAD, U.S.A.). A β-actin antibody was used as a control for whole-cell lysates.

### 16S ribosomal DNA gene sequencing

Fecal genomic DNA was extracted using QIAamp PowerFecal DNA Kit (QIAGEN). The final DNA concentration and purity were determined using a NanoDrop 2000 UV-vis spectrophotometer (Nanodrop Technologies, Wilmington, DE, USA), and the DNA quality was checked using 1% agarose gel electrophoresis. The V3–V4 hypervariable regions of the bacteria 16S rRNA gene were amplified using primers 338 F (5′- ACTCCTACGGGAGGCAGCAG-3′) and 806 R (5′-GGACTACHVGGGTWTCTAAT-3′) in a thermocycler PCR system (GeneAmp 9700, Applied Biosystems, Foster City, CA, USA). The PCR reactions were conducted using the following program: 3 min of denaturation at 95°C; 27 cycles of 30 s at 95°C, 30 s for annealing at 55°C, and 45 s for elongation at 72°C; and a final extension at 72°C for 10 min. The PCR reactions were performed in triplicate in a 20 μL mixture containing 4 μL of 5 × FastPfu Buffer, 2 μL of 2.5 mM dNTPs, 0.8 μL of each primer (5 μM), 0.4 μL of FastPfu Polymerase, and 10 ng of template DNA. The resulting PCR products were extracted from a 2% agarose gel and further purified using an AxyPrep DNA Gel Extraction Kit (Axygen Biosciences, Union City, CA, USA) and quantified using a QuantiFluor™-ST (Promega, Madison, WI, USA) according to the manufacturer’s protocol.

The purified amplicons were pooled in equimolar amounts and paired-end sequenced (2 × 300 bp) on an Illumina MiSeq platform (Illumina, San Diego, CA, USA) according to the standard protocols by Majorbio Bio-Pharm Technology Co. Ltd. (Shanghai, China). The raw reads were deposited in the NCBI Sequence Read Archive (SRA) database (Accession Number: SRP277482).

Raw fastq files were quality-filtered by Trimmomatic and merged by FLASH with the following criteria: (i) The reads were truncated at any site receiving an average quality score < 20 over a 50 bp sliding window. (ii) Sequences whose overlap was longer than 10 bp were merged according to their overlap with a mismatch of no more than 2 bp. (iii) Sequences of each sample were separated according to barcodes (exactly matching) and Primers (allowing two nucleotide mismatches), and reads containing ambiguous bases were removed.

Operational taxonomic units (OTUs) were clustered with a 97% similarity cutoff using UPARSE (version 7.1 http://drive5.com/uparse/) with a novel ‘greedy’ algorithm that performs chimera filtering and OTU clustering simultaneously. The taxonomy of each 16S rRNA gene sequence was analyzed using the RDP Classifier algorithm (http://rdp.cme.msu.edu/) against the Silva (SSU123) 16S rRNA database, using a confidence threshold of 70%.

### *Fecal DNA extraction and* P. copri *quantification*

Fecal genomic DNA was extracted using a QIAamp DNA Mini Kit (QIAGEN) according to the manufacturer’s instructions. Real-time PCR was performed to detect the *P. copri* level using 20 ng of genomic DNA on an Applied Biosystems 7900 quantitative PCR system. *P. copri* quantitation was measured relative to the 16s rDNA gene.

### RNA Extraction and Quantitative Real-time Reverse Transcription PCR

Total RNA was extracted from mouse colonic epithelium cells using the TRIzol reagent and from human colorectal tissues using AllPrep DNA/RNA Mini Kit (QIAGEN) following the manufacturer’s guidelines. Total RNA (1 μg) was reverse transcribed using the PrimeScript RT Reagent Kit (Perfect Real Time; Takara, Shiga, Japan) to detect relative mRNAs. Quantitative real-time PCR was performed in triplicate on an Applied Biosystems 7900 quantitative PCR system as described above. The Ct values obtained from different samples were compared using the 2^−ΔCt^ method. *ACTB* (beta actin) served as the internal reference gene.

### RNA-seq

Samples were sequenced in an Illumina HiSeq 3000 instrument for 2 × 150-bp paired-end sequencing. Reads were mapped to the human genome (hg19) using TopHat v2.0.117 (http://tophat.cbcb.umd.edu) with the default options with a TopHat transcript index built from Ensembl_GRCh37.^57^ Transcript expression was estimated with an improved version of Cuffdiff (http://cufflinks.cbcb.umd.edu). Cuffdiff was run with the default options against the UCSC iGenomes GTF file from Illumina (http://cufflinks.cbcb.umd.edu/igenomes.html). The workflow used to analyze the data was described in detail in Trapnell et al.^58^ To identify a gene or transcript as differentially expression, Cuffdiff2 tests the observed log-fold-change in its expression against the null hypothesis of no change (i.e., the true log-fold-change is zero). Clustering of gene expression profiles was achieved using the csDendro function from CummeRbund (http://compbio.mit.edu/cummeRbund/). The RNA sequence data has been deposited in NCBIs Gene Expression Omnibus (GEO, http://www.ncbi.nlm.nih.gov/geo/) and is accessible through GEO Series accession number GSE156241 (reviewer access token: sfwpqmoqpzchraz)

### Statistical Analysis

Statistical analyses were carried out using the program R (www.r-project.org). Data from at least three independent experiments performed in multiple replicates are presented as the means ± SD. Error bars in the scatterplots and the bar graphs represent the SD. Data was examined to determine whether they were normally distributed using the One-Sample Kolmogorov-Smirnov test. If the data was normally distributed, comparisons of measurement data between two groups were performed using an independent sample t test. If the results showed a significant difference, when the data presented a skewed distribution, comparisons were performed using a nonparametric test. Measurement data between two groups was compared using nonparametric Mann–Whitney U test or Wilcoxon’s rank-sum test. Spearman correlation analysis was performed to determine the correlation between two variables. Fisher’s exact test was used to analyze the pathological grade between two groups. One-way ANOVA was performed to test the difference in *P. copri* numbers in the fecal content between the mice. Two-way ANOVA was used to analyze the body weight and tumor distribution between two groups.

## Supplementary Material

Supplemental Material
