# Supplementary material for: ZFP90 drives the initiation of colitis-associated colorectal cancer via a microbiota-dependent strategy
Source: Gut Microbes. 2021 May 5;13(1):1917269. doi: 10.1080/19490976.2021.1917269 (PMC8115455; doi:10.1080/19490976.2021.1917269)
Supplement: Supplemental Material [file KGMI_A_1917269_SM5328.zip › Supplementary information/Revised Supplementary Figure legends.docx]

**Supplementary Figure 1**

**a** Heatmap of Different gene expressions from colon epithelium in AOM-DSS treated or untreated WT mice. Gene expressions were determined by mRNA array. **b** Relative *Zfp90* expression from colon epithelium in AOM-DSS treated or untreated WT mice (GSE44904). **c** Representative image of H&E staining from *Zfp90*^fl/fl^ and *Zfp90*^△IEC^ mice. **d** Schematic diagram of AOM-DSS induced CAC model. **e** Tumor numbers in *Zfp90*^fl/fl^ and *Zfp90*^ΔIEC^ mice (n = 8 per group). **f** Representative images of spleen from *Zfp90*^fl/fl^ and *Zfp90*^△IEC^ mice. **g** The body weight of *Zfp90*^fl/fl^ and *Zfp90*^ΔIEC^ mice was recorded throughout the acute colitis experiment and was expressed as the ratio relative to the initial weight before DSS treatment (n = 5 per group). **h** Colon length in *Zfp90*^fl/fl^ and *Zfp90*^ΔIEC^ mice at the end of the acute colitis experiment (n = 5 per group). **i** Real-time PCR was performed to determine the mRNA level of *Cdh1* from colons of *Zfp90*^fl/fl^ and *Zfp90*^△IEC^ mice with or without AOM-DSS treatment (n = 5 or 6 per group). **j** Representative data showing protein level of CDH1 in colon tissues from *Zfp90*^fl/fl^ and *Zfp90*^△IEC^ mice with or without AOM-DSS treatment.

**Supplementary Figure 2**

**a** Intestinal permeability assessed by FITC-dextran in DSS-treated *Zfp90*^fl/fl^ and *Zfp90*^ΔIEC^ mice (n = 5 per group). **b** Serum levels of LPS in DSS-treated *Zfp90*^fl/fl^ and *Zfp90*^ΔIEC^ mice (n = 5 per group). **c** Real-time PCR was performed to detect the mRNA level of *Zo-1* from colons of *Zfp90*^fl/fl^ and *Zfp90*^△IEC^ mice with DSS treatment (n = 5 per group). **d** Principal component analysis plot based on RNA-seq of colon epithelium from *Zfp90*^fl/fl^ and *Zfp90*^ΔIEC^ mice upon AOM-DSS-treatment (n = 3 per group). **e** KEGG annotation of 27 different pathways in *Zfp90*^fl/fl^ and *Zfp90*^ΔIEC^ mice with AOM-DSS treatment. **f** Statistical analysis of immunohistochemical immunoreactive score system (IRS) scores of Ly6G and F4/80 in *Zfp90*^fl/fl^ and *Zfp90*^△IEC^ mice (n = 5 per group), Mann-Whitney U test.

**Supplementary Figure 3**

**a** Real-time PCR was performed to detect 16srDNA abundance from H_2_O-treated and antibiotic-treated mice (n = 5 per group). **b** Statistical analysis of immunohistochemical immunoreactive score system (IRS) scores of Ly6G and F4/80 in *Zfp90*^fl/fl^ and *Zfp90*^△IEC^ mice with antibiotic and AOM-DSS treatment (n = 5 per group), Mann-Whitney U test. **c** Schematic diagram of FMT experiment followed by AOM-DSS treatment. **d** Statistical analysis of immunohistochemical immunoreactive score system (IRS) scores of Ly6G and F4/80 in WT mice with FMT from *Zfp90*^fl/fl^ and *Zfp90*^△IEC^ mice (n = 5 per group), Mann-Whitney U test. **e** The body weight of *Zfp90*^fl/fl^ and *Zfp90*^ΔIEC^ mice pretreated with antibiotic was recorded throughout the acute colitis experiment and was expressed as the ratio relative to the initial weight before DSS treatment (n = 5 per group). **f** Colon length in *Zfp90*^fl/fl^ and *Zfp90*^ΔIEC^ mice pretreated with antibiotic at the end of the acute colitis experiment (n = 5 per group). **g** Intestinal permeability assessed by FITC-dextran in antibiotic and DSS-treated *Zfp90*^fl/fl^ and *Zfp90*^ΔIEC^ mice (n = 5 per group). **h** Real-time PCR was performed to detect the mRNA level of *Zo-1* from colons of *Zfp90*^fl/fl^ and *Zfp90*^△IEC^ mice with antibiotic and DSS treatment (n = 5 per group).

**Supplementary Figure 4**

**a** ANOSIM/Adonis analysis was used to test whether the differences between groups are significantly greater than the differences within groups. **b, c** Shannon index and Sobs index based on bacterial 16S ribosomal DNA gene sequence of fecal content from *Zfp90*^fl/fl^ and *Zfp90*^ΔIEC^ mice before and after AOM-DSS-treatment. **d** KEGG annotation of key different metabolic pathways in gut microbes from *Zfp90*^fl/fl^ and *Zfp90*^ΔIEC^ mice. **e** Schematic diagram of AOM-DSS induced CAC model with or without *P.copri* treatment. **f** The body weight of WT mice gavaged with PBS or *P.copri* was recorded throughout the acute colitis experiment and was expressed as the ratio relative to the initial weight before DSS treatment (n = 5 per group). **g** Colon length of WT mice gavaged with PBS or *P.copri* at the end of the acute colitis experiment (n = 5 per group). **h** Intestinal permeability assessed by FITC-dextran in DSS-treated WT mice gavaged with PBS or *P.copri* (n = 5 per group). **i** Serum levels of LPS in DSS-treated WT mice gavaged with PBS or *P.copri* (n = 5 per group). **j** Real-time PCR was performed to detect the mRNA level of *Zo-1* from colons of WT mice gavaged with PBS or *P.copri* after DSS treatment (n = 5 per group).

**Supplementary Figure 5**

**a-c** Real-time PCR was performed to determine the mRNA level of *Pi3k*, *Akt* and *Nfκb* from colons of *Zfp90*^fl/fl^ and *Zfp90*^△IEC^ mice with AOM-DSS treatment.
