# Supplementary material for: ZFP90 drives the initiation of colitis-associated colorectal cancer via a microbiota-dependent strategy
Source: Gut Microbes. 2021 May 5;13(1):1917269. doi: 10.1080/19490976.2021.1917269 (PMC8115455; doi:10.1080/19490976.2021.1917269)

Supplementary Figure 1

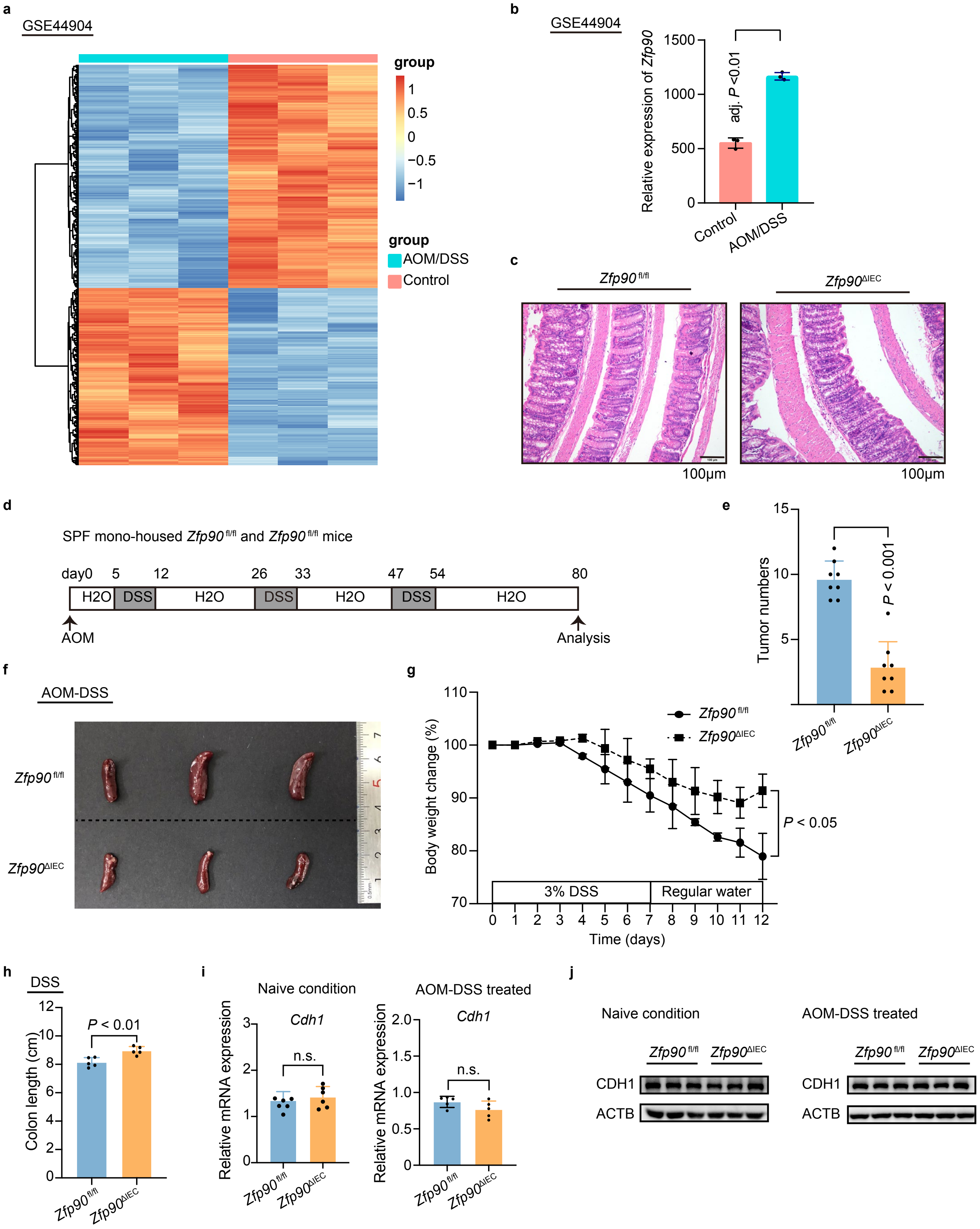

Supplementary Figure 2

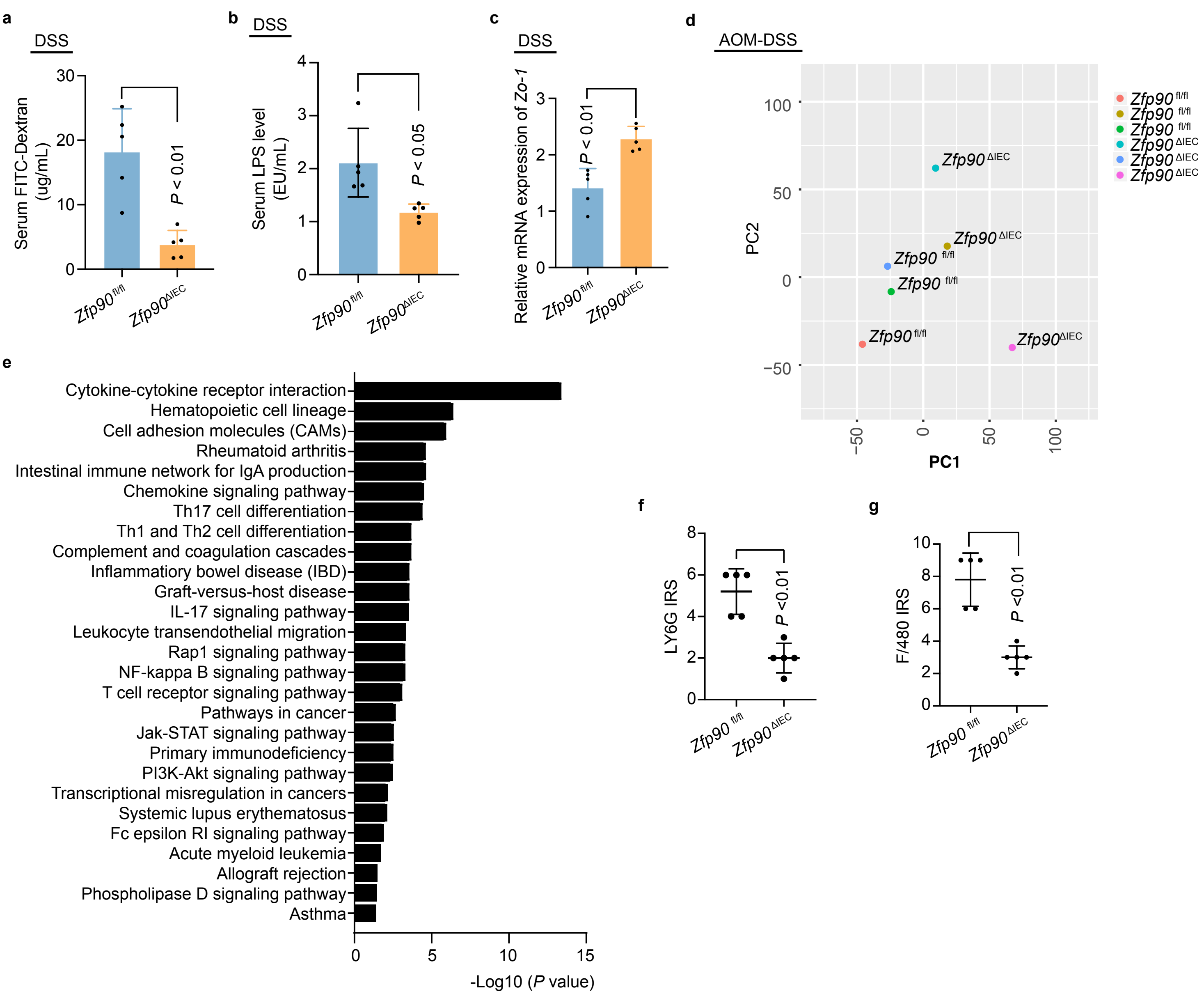

Supplementary Figure 3

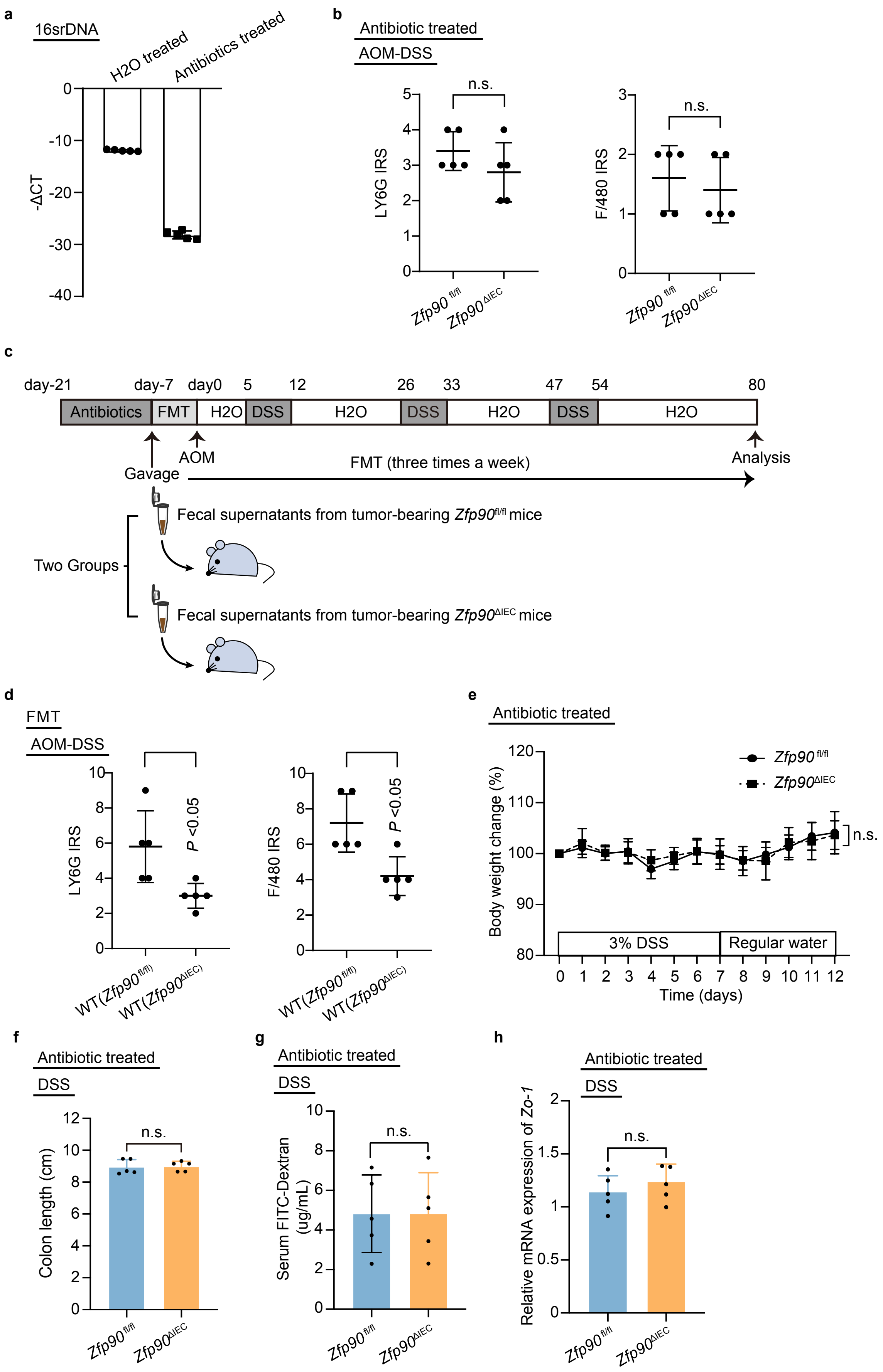

Supplementary Figure 4

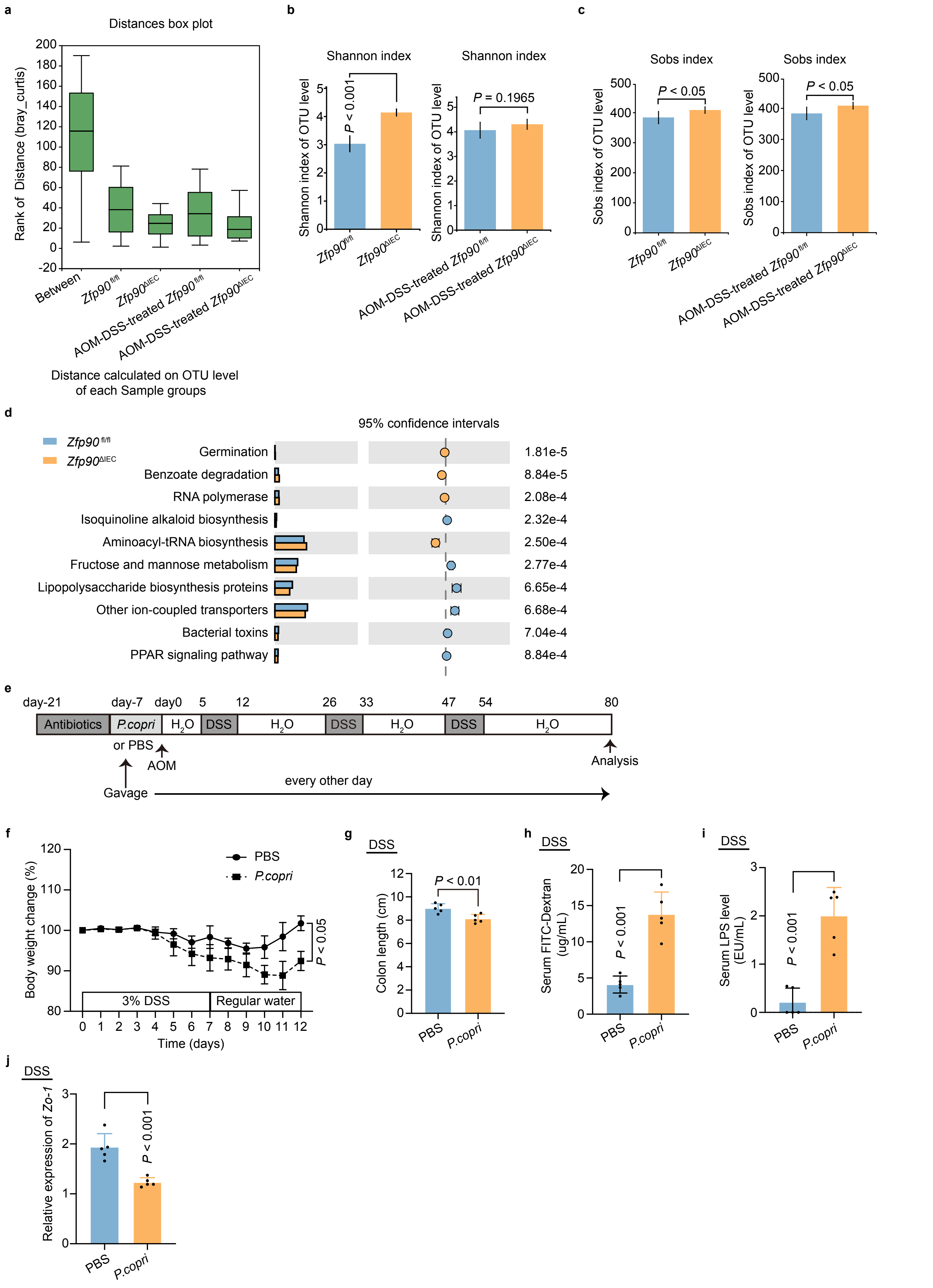

Supplementary Figure 5

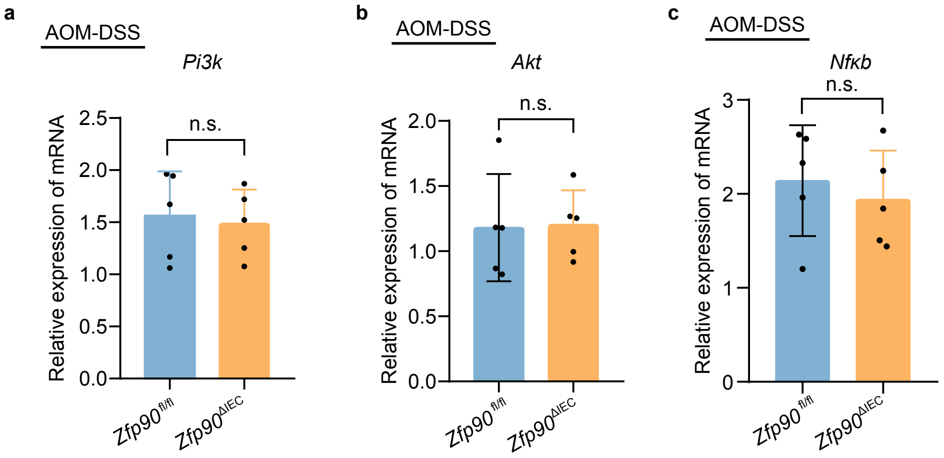

Supplement: Supplemental Material [file KGMI_A_1917269_SM5328.zip › Supplementary information/Supplementary figures.pdf]
